# Introduction of the snake antenna array: Geometry optimization of a sinusoidal dipole antenna for 10.5T body imaging with lower peak SAR

**DOI:** 10.1002/mrm.28297

**Published:** 2020-05-05

**Authors:** Bart Steensma, Pierre‐Francois van de Moortele, Arcan Ertürk, Andrea Grant, Gregor Adriany, Peter Luijten, Dennis Klomp, Nico van den Berg, Gregory Metzger, Alexander Raaijmakers

**Affiliations:** ^1^ Center for Image Sciences University Medical Center Utrecht Utrecht the Netherlands; ^2^ Center for Magnetic Resonance Research University of Minnesota Minneapolis USA; ^3^ Restorative Therapies Group Medtronic Minneapolis USA; ^4^ Biomedical Image Analysis Eindhoven University of Technology Eindhoven the Netherlands

## Abstract

**Purpose:**

To improve imaging performance for body MRI with a local transmit array at 10.5T, the geometry of a dipole antenna was optimized to achieve lower peak specific absorption rate (SAR) levels and a more uniform transmit profile.

**Methods:**

Electromagnetic simulations on a phantom were used to evaluate the SAR and $B_{1}^{+}$‐performance of different dipole antenna geometries. The best performing antenna (the snake antenna) was simulated on human models in a 12‐channel array configuration for safety assessment and for comparison to a previous antenna design. This 12‐channel array was constructed after which electromagnetic simulations were validated by $B_{1}^{+}$‐maps and temperature measurements. After obtaining approval by the Food and Drug Administration to scan with the snake antenna array, in vivo imaging was performed on 2 volunteers.

**Results:**

Simulation results on a phantom indicate a lower SAR and a higher transmit efficiency for the snake antenna compared to the fractionated dipole array. Similar results are found on a human body model: when comparing the trade‐off between uniformity and peak SAR, the snake antenna performs better for all imaging targets. Simulations and measurements are in good agreement. Preliminary imaging result were acquired in 2 volunteers with the 12‐channel snake antenna array.

**Conclusion:**

By optimizing the geometry of a dipole antenna, peak SAR levels were lowered while achieving a more uniform transmit field as demonstrated in simulations on a phantom and a human body model. The array was constructed, validated, and successfully used to image 2 individuals at 10.5T.

## 1 INTRODUCTION

The development of ultra‐high field MRI (UHF, B_0_ ≥ 7T) has been driven by the promise of strongly increasing signal‐to‐noise ratio with increasing B_0_‐field strength.^1^, ^2^, ^3^, ^4^, ^5^, ^6^ Increased chemical shift separation and enhanced susceptibility contrast are additional benefits of UHF MRI. Although the benefits have been primarily presented in the human head, applications below the head in the human torso have been demonstrated. Applications in the body at UHF have paralleled key developments in the development of radiofrequency (RF) coils.^7^, ^8^, ^9^, ^10^, ^11^, ^12^, ^13^, ^14^, ^15^ Since the first preliminary results of body imaging at 7T,^7^ the preferred RF transmit coil design has moved from a traditional whole‐body birdcage resonator used commonly at 3T to local transmit coil arrays driven by multiple RF amplifiers as part of a parallel transmit system where multiple amplifiers are used to drive up to 64 separate transmit elements that can be excited with individually controlled waveforms.^16^, ^17^, ^18^, ^19^, ^20^ A wide variety of different RF coil designs have been developed and tested for MRI. Resonant structures that are commonly used as transmit/receive elements include loop coils, strip‐lines, and dipole antennas. The use of the dipole antenna is becoming increasingly popular at UHF due to the high penetration depth and the relatively uniform transmit field of these elements.^8^, ^11^, ^21^, ^22^, ^23^, ^24^, ^25^

In 2014, the first whole‐body 10.5 T human scanner was installed at the Center for Magnetic Resonance Research in Minneapolis, Minnesota. The first RF coil designed and approved for human studies on this system was for body imaging and consisted of a local transmit array based on fractionated dipole antennas as presented by Ertürk et al.^25^ With this body array at 10.5T, an increase in signal‐to‐noise ratio of more than 2‐fold (2.26) was experimentally demonstrated in the center of a body‐sized phantom compared to 7.0 T^25^ and was later demonstrated to yield the first human body images at 10.5T.^26^ The 2.26‐fold increase indicates that signal‐to‐noise ratio for body MRI increases quadratic with B_0_ field strength from 7T to 10.5T, which is in line with simulation results on a human model of the prostate.^27^ An in vivo comparison of signal‐to‐noise ratio in multiple subjects as in Pohman et al^28^ is not yet available. However, simulations on a human body model indicated that 10g averaged peak local specific absorption rate (SAR_10g_) for a given excitation fidelity and number of pulses was always higher at 10.5 T than at 7.0 T. Simulation results in the brain^29^ and in the prostate^27^ indicate that between 7T and 14T, 10g averaged peak local SAR increases stronger than linear with B_0_‐field strength. Especially at UHF, peak local SAR_10g_ is a limiting factor for acquisition speed and it is crucial to manage peak local SAR to maintain imaging performance. Given that peak local SAR is already a limiting factor at 7.0 T, devising strategies to address the increasing peak local SAR at 10.5 T becomes a critical issue to solve.

The geometry of a dipole antenna strongly influences the magnetic and electric fields that it emits.^22^, ^23^ The peak SAR_10g_, the $B_{1}^{+}$ per unit power (transmit efficiency), and the $B_{1}^{+}$ per unit peak SAR_10g_ (SAR efficiency) depend on dipole antenna geometry. Previously, a sinusoidal geometry, henceforth referred to as the snake antenna, has shown desirable characteristics in 7T body imaging applications.^30^ In this work, we present the optimization of the snake antenna’s geometry to obtain maximum SAR efficiency for body imaging at 10.5 T. Results from the RF array validation process and in vivo imaging at 10.5T are demonstrated in human volunteers as part of a Food and Drug Administration (FDA) Investigational Device Exemption safety study.^31^

## 2 METHODS

### 2.1 Antenna geometry optimization

**FIGURE 1:**
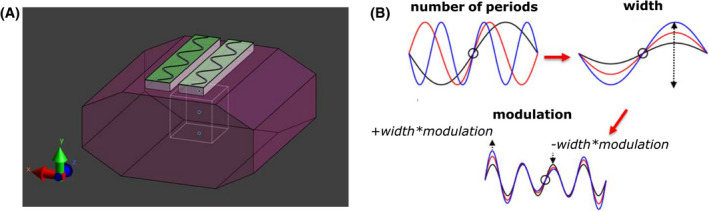
The optimization setup containing 2 dipole antennas (center‐to‐center distance 6 cm), on a phantom with tissue mimicking dielectric properties (ε_r _= 0.34, σ = 0.4 S/m). The number of periods, the width, and the modulation of the snake dipole were varied. $B_{1}^{+}$ was calculated in a 10 × 10 × 10 cm^3^ cubical region of interest (ROI), centered at 10 cm depth

Finite difference time‐domain simulations (Sim4Life version 3.4, Zurich Medtech, Zurich, Switzerland) were used to model 2 sinusoidally shaped dipole antennas on a phantom with tissue‐like properties (σ = 0.37 S/m ε_r _= 34, width = 41 cm, height = 20 cm, length = 38 cm). The geometry of the dipole antennas was varied by changing the number of periods, the width, and the modulation of the sinusoidal geometry (Figure 1). The optimization was done sequentially. After finding the optimal number of periods, the optimal width was found for the optimal width and after that the optimal modulation was found for the optimal number of periods and width. Modulation was implemented as follows: for a modulation of 0.5, the width of the antenna was 1+0.5*width at the distal end of the antenna and 1‐0.5*width at the center of the antenna. This is indicated in Figure 1.

The longitudinal extent of the dipole antennas was kept equal to the length of the fractionated dipole antenna for 10.5T in all cases. Phase shimming was applied on a cubic region of interest (ROI, 10 cm^3^) located at a depth of 10 cm in the phantom. The different antenna geometries were evaluated by calculating the 10g‐averaged peak SAR^32^ and by assessing $B_{1}^{+}$ in the ROI. The optimum antenna was selected based on the highest $B_{1}^{+}$/√SAR_10g_ in the ROI.

### 2.2 Antenna array simulations

To determine safe limits of operation according to the International Electrotechnical Commission guidelines, electromagnetic simulations were done in Sim4Life to assess peak local SAR levels for the snake antenna array. A 12‐channel snake antenna array was simulated on the human models Duke and Ella^33^ for 4 different imaging targets: prostate (Duke), uterus (Ella), heart, and kidneys. Material properties of the human models were taken from Sim4Life, based on the work of Gabriel et al.^34^ An adaptive grid size was used, with a minimum grid size of 1.5 mm^3^ for voxelization of conductors, sources, and lumped elements. This resulted in a grid size of at maximum 20 × 10^6^ cells, with a simulation time of approximately 20 minutes per port on a GPU (Tesla K20, Nvidia, Santa Clara, California). Every simulation ran for 30 periods or until a convergence of 50 dB was achieved. All antennas were positioned with a minimum antenna‐subject distance of 20 mm. Electric and magnetic fields were exported to Matlab (Mathworks, Natick, Massachusetts) and resampled to a 5 mm isotropic resolution to keep SAR calculations tractable. A post‐processing script was used to calculate 10g averaged Q‐matrices^35^ and to calculate generalized virtual observation points,^36^, ^37^ (overestimation percentage 1%). For all shim targets, 2 shim drives were calculated that respectively maximized the mean $B_{1}^{+}$ or minimized the coefficient of variation (standard deviation divided by the mean) in the imaging target. Both phase and amplitude were optimized using the Matlab function fmincon with constraints on maximum input power (1W per channel). The final SAR values were normalized to a total input power of 1W. After the 2 shim drives were known, peak SAR was calculated for 10,000 random shim vectors within the range spanned by these 2 RF shim drives to calculate peak SAR values for a distribution of realistic shimming scenarios. The resulting distributions of peak SAR values were plotted in a histogram to which a gamma distribution was fitted. Based on the normal distribution, the SAR value was found that had a chance of being exceeded by less than 0.1% (the pSAR_99_ value, as presented by Meliado et al^38^).

To compare the performance of the snake antenna array with a previous design^25^ in a full array setup, two 12‐channel setups were compared on the Duke model: a setup consisting of fractionated dipole antennas and a setup consisting of the snake antennas. To investigate coil performance in terms of uniformity and peak local SAR for different shim settings, L‐curves were calculated for the fractionated dipole array and the snake antenna array on all imaging targets for Duke. A multishift conjugate gradient algorithm was used to minimize the following least squares optimization problem:

$$\arg\min_{x}\left({\left|\left|Ax-m\right|\right|}^{2}+\lambda \left|\right|{\left|x\right|}^{2}+\eta \sum_{j}\alpha_{j}{\left|\left|S_{j}x\right|\right|}^{2}\right)$$

A is a matrix containing $B_{1}^{+}$ distributions for every channel, m is a vector containing the target $B_{1}^{+}$ (1 μT over the shimming ROI), x is a vector of complex coil weights, and S_j_ is a sparse matrix containing the virtual observation points for peak local SAR calculation. The minimization was done for different values of the regularization η and could be done simultaneously for different values of λ. After finding an optimal value for η, L‐curves are plotted for the different values of λ.

### 2.3 Implementation of snake antenna array

An array of 12 snake antennas was built for validation and to conduct imaging studies. The dipole conductors were etched on a single‐sided FR4 printed circuit board (EuroCircuits Gmbh, Kettenhausen, Germany). The dipoles were placed on 10‐mm thick thermoplastic polyetherimide (ULTEM 1000 resin, Sabic Global, Pittsfield, Massachusetts) blocks. ULTEM blocks 5 mm thick were placed on top of the Printed Circuit Board to cover the conductors. The dipole antennas were tuned by 2 series capacitors (1 pF) and matching was achieved by a lattice balun. All channels were matched to −12 dB reflection or less. For a center‐to‐center distance of 6 cm, interelement coupling was lower than −13 dB.

### 2.4 Experimental safety assessment

All imaging experiments were done on a Siemens Magnetom whole‐body 10.5T system (Siemens Healthineers, Erlangen, Germany). The scanner has a 32‐channel receive setup and a 16‐channel “Step 2” parallel transmit system for RF transmission. The transmit channels were driven by 2‐kW power amplifiers (Stolberg HF‐Technik AG, Stolberg, Germany). The whole‐body SC 72 gradient systems (Siemens Healthineers) can achieve a maximum amplitude of 70 mT/m and a slew rate of 200 T/m/s. For validation of the simulations, $B_{1}^{+}$‐maps (actual flip‐angle method, resolution 2.5 × 2.5 × 2.5 mm^3^, pulse repetition time (TR) 20/120 ms,^12^ acquisition time 8:00 minutes) and Magnetic Resonance Thermometry maps (Proton‐Resonance Frequency‐shift method,^39^ TR 7.5 ms, input voltage 120 V, duty cycle 6.67%, total heating time 4 × 4:00 minutes) were acquired experimentally. The duty cycle of 6.67% and input voltage of 120 V were used to achieve a time averaged input power of 19.2 W/channel within the hardware limitations of the RF amplifiers. This duty cycle and average power were necessary to heat the phantom sufficiently for improvement of the performance of magnetic resonance thermometry measurements. The experiments were performed with a body size phantom^40^ filled with a hydroxyethyl cellulose solution containing 2.97 g NaCl/L and 14 g hydroxyetheyl cellulose/L, specific heat capacity C_p_ of 3178 $\frac{J}{\mathrm{kgK}}$. To scale the measured temperature maps to SAR maps, a linear scaling was applied according to $\left(SAR_{10g}=C_{p}\frac{\Delta T}{\Delta t}\right)$. As the FDA considers the RF coils part of the MRI system and because the MRI scanner was >8.0T, the FDA requires validation of all coils prior to in vivo use. The validation is made based on data presented in this manuscript closely following previously published strategies.^41^ Briefly, for comparison with simulation, experimental $B_{1}^{+}$ maps were acquired with actual flip‐angle imaging and SAR maps were determined from Magnetic Resonance Thermometry measurements during separate heating studies the results of which are presented in this paper. Validation of SAR simulations can be done through MR thermometry, or through fiber optic probe measurements as done in He et al.^26^ Validation measurements were first done for a single channel of both the snake and fractionated dipole antennas, and then for the complete 12‐channel arrays, both of which were subsequently compared to simulations. For the measurements with the full array, 2 RF shims were included in the comparison: an RF shim where all transmit phases were 0° and an RF shim where odd/even channels had a transmit phase of 0°/180°. These RF shims were picked because of their distinctly different SAR profiles. To determine potential scaling factors between the simulations and the measurements, single channel B1‐maps were acquired for all channels, the simulated data were co‐registered against these measured B1‐maps. The ratio between the 2 was determined in regions were the measured B1‐map showed values within the acceptable range of the measurement method. Based on the ratio between the single channel B1‐maps, scaling factors were determined for every transmit channel, which were applied on the multichannel simulations. The simulated and measured B_1_‐ and SAR‐maps were normalized to 1W total input power while accounting for the measured losses in the transmit chain. The simulated and measured multichannel $B_{1}^{+}$‐ and SAR‐maps were co‐registered, after which spatial correlation was calculated between the simulated and measured maps for an ROI covering the completer center slice of the phantom.

### 2.5 In vivo imaging

Subjects provided written signed consent to participate in an FDA and institutional review board approved Investigational Device Exemption safety study primarily investigating effects of static field exposure.^31^ The 2 subjects imaged with the snake antenna array included a 43‐year‐old female (89 kg, 180 cm, abdomen imaging) and a 41‐year‐old male (86 kg, 178 cm, prostate imaging). For the pelvis imaging experiments, anatomical scout images (T1w 2D gradient echo, TR/echo time [TE] = 10/3.61 ms, resolution 1.56 × 1.56 × 5 mm^3^, FA 10°, acquisition time 0:06 s) and B1‐maps are shown (actual flip‐angle imaging method, TR/TE = 70/3 ms, resolution 2.73 × 2.73 × 8 mm^3^, FA 50°, acquisition time 6:44 s). These images are shown as well for the abdominal study, where additionally a T1 weighted anatomical image of the kidneys is shown (Fat‐saturated T1w 2D gradient echo, TR/TE 200/3.69 ms 0.83 × 0.83 × 5 mm^3^, FA 18°, acquisition time 0:25 s). All images were acquired during a breath‐hold.

## 3 RESULTS

### 3.1 Antenna geometry optimization

**FIGURE 2:**
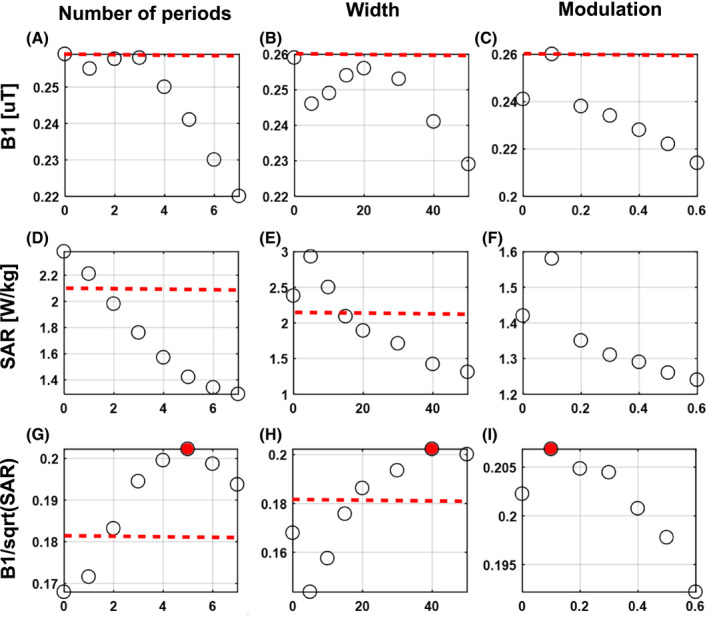
Resulting average $B_{1}^{+}$ in the region of interest (ROI), peak specific absorption rate (SAR)_10g_ in the phantom, and SAR efficiency in the ROI. The effect of number of periods, width, and modulation was optimized sequentially. After finding the optimal number of periods, the optimal width was found for the optimal width and after that the optimal modulation was found for the optimal number of periods and width. Red dashed line indicates performance for the fractionated dipole array. All results were normalized to 1W accepted power on both channels

Increasing the meander width or the number of meanders, which corresponds to an increase in total conductor length, generally decreased transmit efficiency and also decreased SAR. The effects of modulating these parameters and their impact on transmit efficiency, SARmax, and SAR efficiency in a phantom are shown in Figure 2.

**FIGURE 3:**
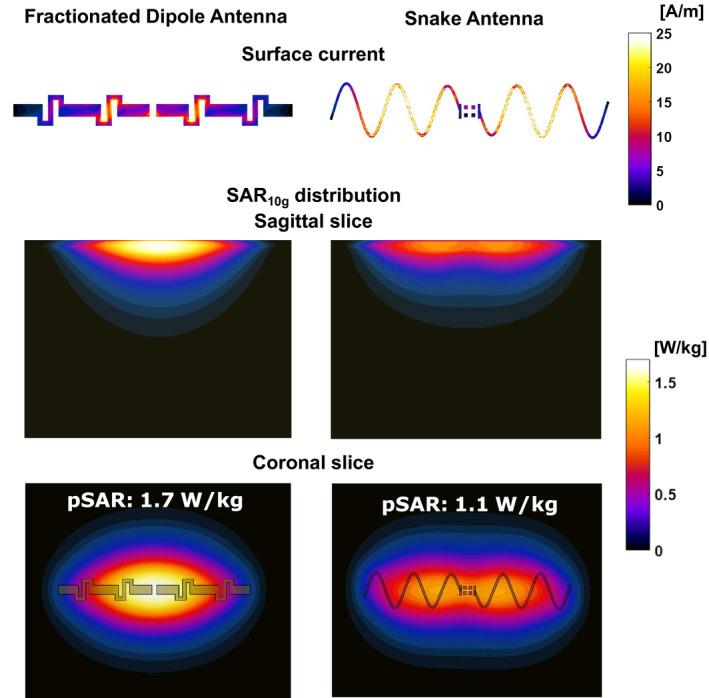
Surface current distribution on a fractionated dipole antenna and on the new design of the snake antenna. The low current at the feed‐point location of the snake antenna results in a lower peak specific absorption rate (SAR) value

When considering SAR efficiency, an optimum can be observed for a number of 5 periods and a meander width of 40 mm. SAR efficiency improves 11% from 0.18 μT/√W/kg for a fractionated dipole to 0.2 μT/√W/kg for the optimal snake antenna design. Modulation of the antenna geometry in general has a less pronounced effect, SAR efficiency ranges between 0.206 and 0.192 μT/√W/kg, where a linear modulation of 0.1 results in the highest SAR efficiency. Figure 3 shows the current distribution together with the resulting SAR distributions for a single channel on a fractionated dipole as compared to the snake antenna. Modifying the conductor geometry removes the high current in the center of the dipole antenna. This high current is also associated with high peak SAR under the center of the dipole antenna, which is an unwanted side effect. Because of the long conductor length, peak SAR_10g_ decreases in accordance with results for long dipole antennas on a phantom.^22^ The effect is a significantly lower SAR for the snake antenna compared to a fractionated dipole.

### 3.2 Antenna array simulations ‐ safety

**FIGURE 4:**
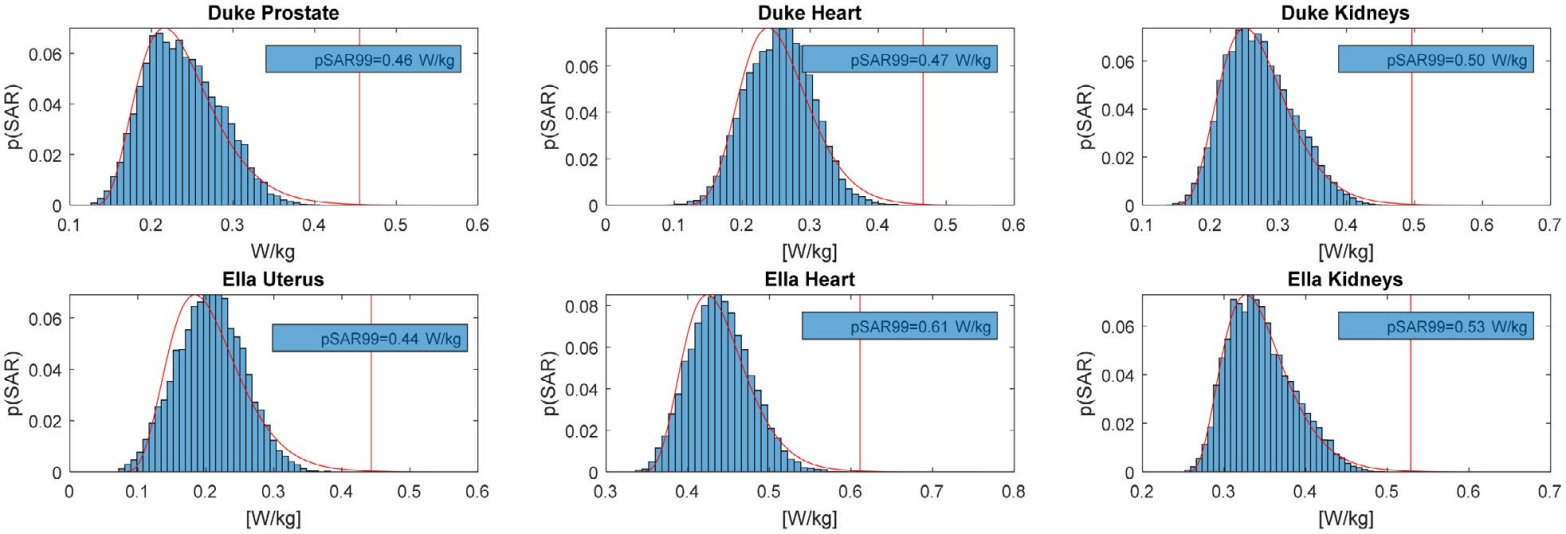
Peak specific absorption rate (SAR) values for different imaging targets, considering radiofrequency (RF) shims that were optimized for uniformity or efficiency in the imaging target and all RF shims within the range shimmed by these two solutions. About 1W total input power was used in all cases. A gamma distribution was fitted to the distribution of peak SAR values, to find a SAR value that has a chance of less than 0.1% of being exceeded for the fitted distribution (pSAR_99_ value). y‐axis represents the probability of occurrence for a given SAR level, where the total probability sums up to 1

For all shim targets, a worst‐case SAR value of 0.47 W/kg is found (Ella, Kidneys) using 1W total input power. Based on an allowed peak SAR level of 20 W/kg, a total average power of 42 W or a per channel average power of 3.5 W/channel would be allowed. In the work of Meliado et al,^38^ a safety factor of 1.8 was found for an 8‐channel dipole array for 7T prostate imaging. Because of the increased number of transmit channels and the increased B_0_ field strength, a conservative safety factor of 3 was added to account for possible intersubject variation, power monitoring errors, and modeling errors, leading to a total average power limit of 1.17 W/channel. Results of the SAR analysis are shown in Figure 4 (1W total input power was used in all cases).

### 3.3 Antenna array simulations – performance

**FIGURE 5:**
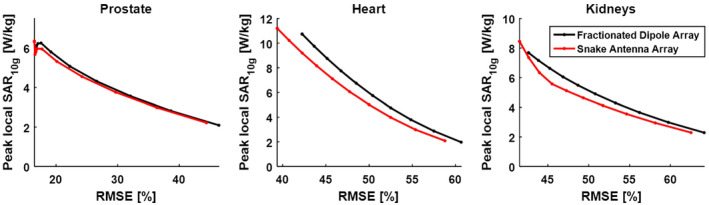
Peak local specific absorption rate (SAR)_10g_ and root mean square excitation error (target $B_{1}^{+}$ 0.5 μT) showing the trade‐off between uniformity and peak SAR for both arrays. For both the heart and the kidneys, lower root mean square error values as well as lower peak SAR values can be achieved with the snake antenna array

Trade‐off curves between root mean square error and peak local SAR indicate that for all imaging targets in this comparison, lower root mean square error values can be achieved with the snake antenna array compared to the fractionated dipole array. For comparable root mean square error values, lower peak SAR values are achieved with the snake antennas. Results of root mean square error versus peak local SAR are plotted for the prostate, heart, and kidneys of Duke in Figure 5.

### 3.4 Experimental safety assessment

**FIGURE 6:**
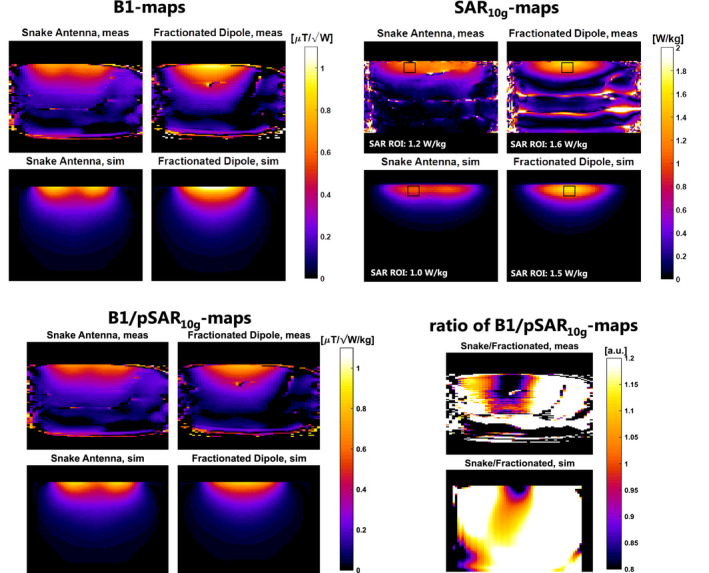
Single channel validation results, showing simulated and measured B1 and specific absorption rate (SAR)_10g_ maps for the fractionated dipole antenna and the snake antenna. Additionally, B1‐maps are shown after normalization for peak SAR_10g_ (SAR efficiency). The ratio of SAR efficiency that the snake antenna has a higher SAR efficiency for locations at depth and away from the center of the antenna. This effect is somewhat stronger in simulation, where B1 coverage is available at depth

Simulated $B_{1}^{+}$‐profiles (Figure 6A) and SAR maps (Figure 6B) were compared to measurements for a single channel of the snake antenna and the fractionated dipole antenna. SAR maps were derived from the temperature measurements by applying a linear scaling ( $SAR_{10g}=C_{p}\frac{\Delta T}{\Delta t}$). The snake antenna has a slightly lower transmit efficiency, especially in the center of the antenna. The simulated and measured transmit efficiency show the same patterns and magnitude (Figure 6A). The same comparison was done for temperature measurements (MR thermometry) and SAR simulations, all results are shown in Figure 6. A lower SAR is achieved with the snake antenna compared to the fractionated dipole antenna. The single channel B1‐maps are scaled with peak SAR to achieve at spatial maps of SAR efficiency (Figure 6C). SAR efficiency of the 2 antennas is compared by dividing the efficiency of the snake antenna by the efficiency of the fractionated dipole antenna. It is observed for both simulations and measurements that the snake antenna has a higher efficiency at locations at depth and away from the isocenter of the antenna. This effect seems more pronounced in the simulations as can be observed in the ratio maps, because the $B_{1}^{+}$ map has a lower accuracy in areas far away from the phantom surface where $B_{1}^{+}$ levels are low. The results of determining the scaling factors from the single channel $B_{1}^{+}$‐maps is added as Supporting Information Figure S1.

**FIGURE 7:**
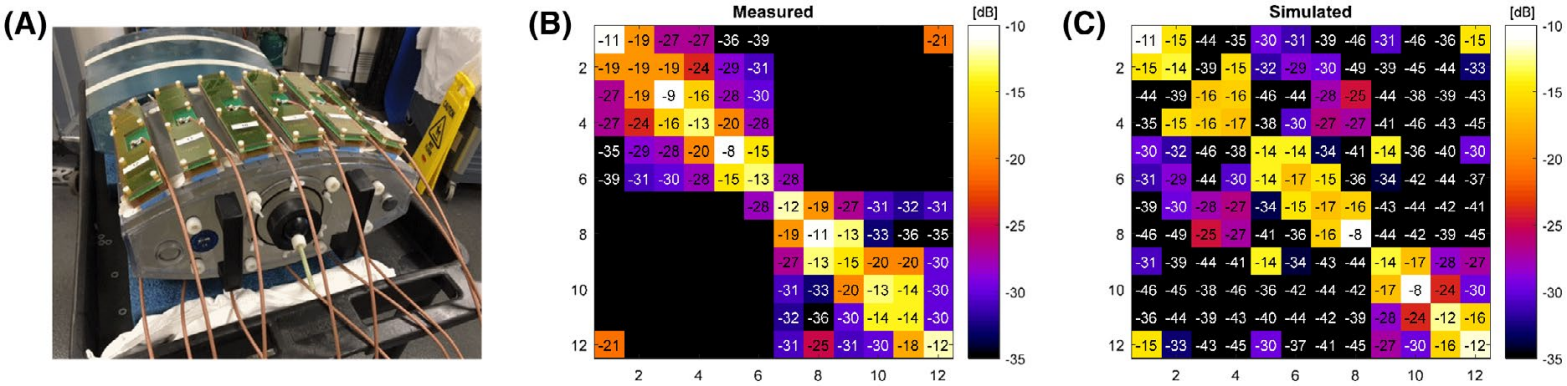
12‐channel snake antenna array on the hydroxyetheyl cellulose phantom (A) Scattering parameters as measured on the bench (B) and as simulated (C). For the bench measurements, scattering parameters were measured only between antennas in the same rows (bottom and top of phantom) and for adjacent antennas (1/12 and 6/7)

An image of the 12‐channel snake antenna array on the validation phantom is shown in Figure 7A. Scattering parameters were measured on the bench (7b) and compared to simulated scattering parameters (7c).

**FIGURE 8:**
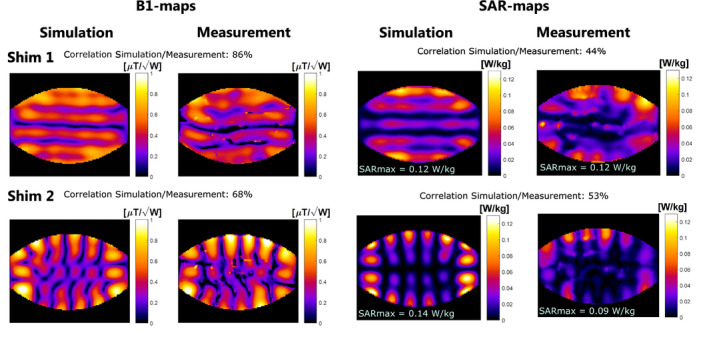
Validation results for the full 12‐channel array configuration. Shim 1 has 0° phase offset for all channels whereas shim 2 has 0°/180° phase offset for odd/even channels. Pearson’s correlation coefficient was calculated between the simulated and measured results

The measured and simulated scattering parameters in Figure 7 are of the same magnitude, the worst‐case interelement coupling is −13 dB in measurements and −14 dB in simulations. Deviations between the measurements and simulations are visible both for the reflection coefficients and for interelement coupling. $B_{1}^{+}$‐mapping and temperature mapping was also done for the 12‐channel array using 2 distinct RF shims: “shim 1” with all transmit phases zero and “shim 2” with alternating 0/π phase offset for odd and even channels. Pearson’s linear correlation coefficient was calculated between the simulated and measured maps. For shim 1, a correlation of 86% was found for the $B_{1}^{+}$‐map and a correlation of 44% was found for the SAR map. For shim 2, a correlation of 68% was found for the $B_{1}^{+}$‐map and 53% for the SAR map. For both shims, the simulated maximum SAR did not exceed the SAR derived from temperature measurements (Figure 8).

### 3.5 In vivo imaging results

**FIGURE 9:**
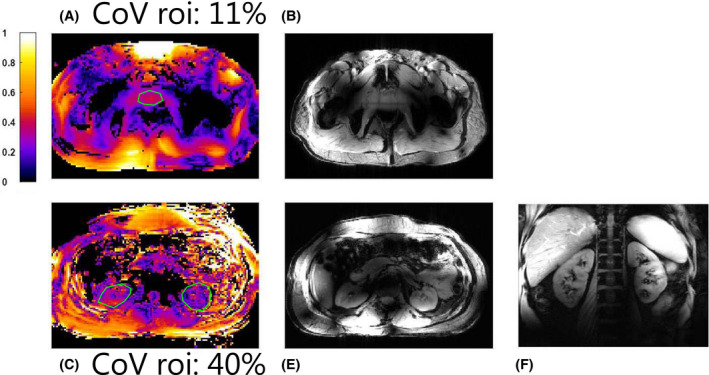
In vivo image of the pelvis (top row) and abdomen (bottom row). B1‐maps (A and C) were acquired for both anatomies using the actual flip‐angle imaging method, as well as scout images (B and D). For the kidneys, a fat suppressed coronal image is shown (E)

After validating the simulation results with measurements and obtaining institutional review board and FDA approval, 2 volunteers were scanned using the snake antenna array. The results are shown in Figure 9.

Figure 9A shows a B1‐map on the prostate. The coefficient of variation (standard deviation divided by average of $B_{1}^{+}$) was calculated in the prostate. A coefficient of variation of 11% was achieved, indicating that a uniform flip angle can be achieve throughout the prostate. This is reflected in the uniformity of the scout image. For kidney imaging, a lower and less uniform $B_{1}^{+}$ is obtained in the kidneys (coefficient of variation 40%). This can be observed in the transverse scout image, where a nonuniform image intensity is visible in the kidneys. However, in the coronal anatomical image, signal intensity remains more uniform throughout the kidneys.

## 4 DISCUSSION

This work demonstrates a comprehensive method for optimizing the transmit coil geometry based on a 2 port simulation on a phantom. The optimization results indicate that dipole antennas with a longer total conductor length (increased number of periods or increased width) not only have a lower SAR but also a lower transmit efficiency. Similar results were obtained in earlier work for fractionated dipole antenna with increased length. An optimum is found for an antenna with 5 periods, a conductor width of 40 mm and a modulation of 0.1. When comparing the field distribution of a single snake antenna with a fractionated dipole antenna, it is observed that SAR is lower and $B_{1}^{+}$ is more evenly distributed in the z‐direction. Close to the surface of the antenna, 2 peaks appear in the electric and magnetic fields, which indicate that a second order mode exists on the dipole antenna. This second order mode has a low current at the feed‐port, resulting in a lower SAR for the snake antenna compared to a fractionated dipole. Transmit efficiency is also lower for the snake antenna, but especially for deeply situated imaging targets this effect is small. Therefore, a known desirable characteristic of the dipole antenna (ie, a more uniform transmit profile with depth^23^, ^24^) is accentuated with the snake antenna design.

Other methods for decreasing SAR of dipole antennas have been reported by different groups, for example, using a passive feed‐point mechanism to lower SAR of the fractionated dipole antenna,^42^ increasing the subject‐antenna distance,^43^ increasing the distance between the feed‐point and the subject^44^ or integrating materials with different dielectric properties in the dipole substrate.^45^ Completely different antenna geometries with equivalent current distributions such as meander stripline antennas,^12^, ^15^ a slot antenna^46^ or a dual‐mode dipole antenna^47^ can also be used to lower SAR. A comparison study of these methods at different field strengths in the same simulation setup, or a combination of the methods (ie, snake antennas with increased distance between the feed‐point and the subject) could also be explored but is considered to be beyond the scope of this work where the effect of the dipole geometry itself is considered rather than the use of different or multiple antenna types.

The method used in this work to find an optimal dipole antenna geometry made use of only 2 channels and a phantom, which deviates from the configuration in which the antennas are used (12 channels on a human subject). This simple optimization procedure is validated as the snake antenna array performs better than the fractionated dipole array when used in the full 12‐channel configuration. Recent studies have considered dipole inductance, length,^48^ and topology^49^ in a full array setup on phantom and a head model, respectively. Improvements in performance can be expected when optimizing the snake antenna using such an approach; however, this is a very time intensive process. Increasing the number of channels from 2 to 12 increases the simulation time 6‐fold. The increase in number of voxels that would be necessary for a full array causes an additional expected increase in simulation time of the same order of magnitude. The introduction of faster electromagnetic simulation methods^50^ could resolve this problem by enabling the rapid calculation of many more antenna configurations making this approach more practical for converging to an optimal array design.

The validation procedure shows a very good agreement between field patterns observed for the measurements with a single dipole antenna. The simulated and measured scattering matrices are of the same order of magnitude but deviations are observed for the reflection coefficients as well as for interelement coupling, even though the same tuning capacitors were used in the simulations and the measurements. Differences between the simulated and measured scattering parameters could be the result of deviations between real and simulated electrical properties of the phantom or the antenna substrates, or possibly stair casing effects in the voxelization of the snake antenna conductors. For the multichannel MRI measurement, a moderately high correlation is found between measurements and simulations. Measurements especially deviate from the simulations at the side of the phantom. It is expected that the matching between simulations and measurements can be improved by measuring the scattering parameters of the array in the MRI scanner using bidirectional couplers, which was not possible at the time the experiments were performed for this work. Especially for the antennas on the side of the phantom, strong deviations are found between measurement and simulation that could be the result of coupling to the nearest neighbors and the opposing antennas. The difference between simulations and measurements is especially strong for the SAR comparison, which is likely the result of differences in SAR scaling the quadratic with differences in electric field. To convert measured temperature maps to SAR maps, we have applied a linear scaling factor $SAR_{10g}=C_{p}\frac{\Delta T}{\Delta t}$. This linear conversion introduces errors, as SAR does not necessarily directly correlate to temperature increase. However, it is sufficient to obtain information the location of high SAR voxels and about the validity of SAR simulations.

In this work, for both the simulations and the measurements, only static RF shimming was used to shape the RF transmit field. Strong improvements in image uniformity could be achieved by employing different RF transmit strategies. The Time Interleaved Acquisition of Modes method has been used before to successfully achieve uniform RF excitation in the body at 7T.^51^ Specifically at 10.5T in the body, RF spokes pulses were used to improve image uniformity for kidney and liver imaging.^26^ Because this work focuses on a proof of principle of the hardware aspects and limited time was available to scan the 2 volunteers with the snake antenna array, we omitted an elaborate comparison of RF performance in vivo, but this can be a subject for future work with possibly even more coil design variations included.

## 5 CONCLUSION

The conductor geometry of a sinusoidal dipole antenna was optimized for a 2‐channel setup on a pelvis phantom at 10.5T. Simulations on a human model indicate that with this new antenna design, more uniform transmit field distributions can be achieved with lower peak SAR values over a range of RF shim solutions. Simulations were validated by comparison to $B_{1}^{+}$ maps and temperature maps acquired with the proton resonance frequency shift method. A 12‐channel array of the optimized snake antennas was constructed, validated, and successfully used to perform initial abdominal imaging studies at 10.5T.

## CONFLICT OF INTEREST

Bart Romke Steensma is a minority shareholder of the MRI company WaveTronica B.V.

## Supporting information


**FIGURE S1** Simulated and measured B1‐maps before and after scaling, and the scaling factors that were applied to every channel
