# Supplementary material for: Introduction of the snake antenna array: Geometry optimization of a sinusoidal dipole antenna for 10.5T body imaging with lower peak SAR
Source: Magn Reson Med. 2020 May 5;84(5):2885–96. doi: 10.1002/mrm.28297 (PMC7496175; doi:10.1002/mrm.28297)
Supplement: Supplementary file 1 — FIGURE S1 Simulated and measured B1‐maps before and after scaling, and the scaling factors that were applied to every channel [file MRM-84-2885-s001.docx]

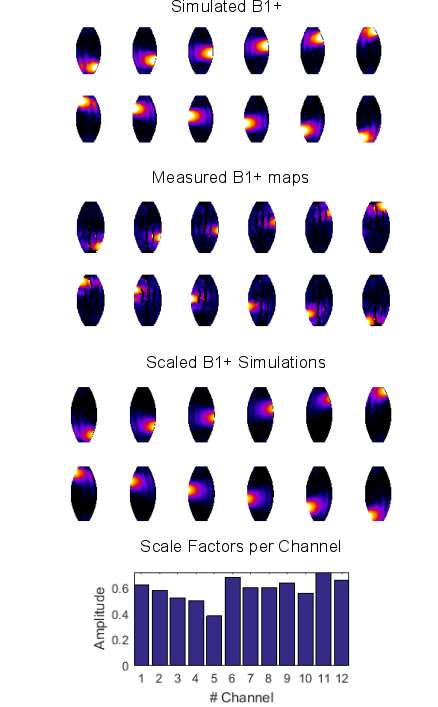


S 1: simulated and measured B1-maps before and after scaling, and the scaling factors that were applied to every channel.
